# Thyroid dyshormonogenesis caused by iodotyrosine deiodinase pathogenic variant: three cases presenting in adolescence

**DOI:** 10.1210/jcemcr/luag111

**Published:** 2026-05-14

**Authors:** Isabelle En Xin Choong, Suet Ching Chen, Mohamad Guftar Shaikh

**Affiliations:** Developmental Endocrinology Research Group, University of Glasgow, Glasgow G12 8QQ, UK; Department of Paediatric Endocrinology, Royal Hospital for Children, Glasgow G51 4TF, UK; Developmental Endocrinology Research Group, University of Glasgow, Glasgow G12 8QQ, UK; Department of Paediatric Endocrinology, Royal Hospital for Children, Glasgow G51 4TF, UK; Developmental Endocrinology Research Group, University of Glasgow, Glasgow G12 8QQ, UK; Department of Paediatric Endocrinology, Royal Hospital for Children, Glasgow G51 4TF, UK

**Keywords:** genetics, congenital hypothyroidism, thyroid dyshormonogenesis, iodotyrosine deiodinase (*IYD*) gene, levothyroxine

## Abstract

We describe three patients from three unrelated consanguineous families, all homozygous for the c.301C>T (p.Arg101Trp) variant in the iodotyrosine deiodinase *(IYD)* gene. These patients presented in late childhood around puberty with diffuse goiter, primary hypothyroidism, and negative thyroid autoantibodies, consistent with thyroid dyshormonogenesis (DH). Initiation of levothyroxine therapy led to a significant reduction in goiter size and clinical improvement. Notably, all patients had normal thyroid function at neonatal screening, suggesting a delayed clinical manifestation of congenital hypothyroidism. These cases highlight the importance of considering genetic causes of hypothyroidism, particularly in consanguineous families. The ultrasound findings of a homogeneously enlarged, hypervascularized goiter without micronodularity in adolescent patients with non-autoimmune hypothyroidism should prompt consideration of DH, specifically *IYD* variants. Increased clinical awareness, combined with targeted molecular testing once an index patient is identified, can facilitate detection of the pathogenic variant, timely initiation of treatment, and screening of at-risk family members.

## Introduction

Hypothyroidism is a condition caused by an underactive thyroid gland, leading to reduced thyroid hormone levels. It can either present at birth (congenital) or develop later in life (usually acquired). Primary congenital hypothyroidism (CH) is the most common congenital endocrine disorder, affecting 1 in 2000-4000 newborns [1]. It arises from either thyroid dysgenesis (TD), developmental defects of the thyroid gland, or thyroid dyshormonogenesis (DH), which results from defects in thyroid hormone synthesis. Genetic causes have only been identified in 5*-*10% of TD cases and 15*-*20% in DH cases [2]. However, these may be underestimations as congenital hypothyroidism may, in some cases, demonstrate oligo- or polygenic inheritance, incomplete penetrance, or somatic mosaicism. For example, incomplete penetrance has been described in association with *PAX8* variants [3], and environmental factors such as nutritional iodine intake may influence phenotypic expression in defects affecting iodide transport (eg, *SLC5A5, SLC26A4*) [4]. Genetic causes of TD and DH present with a broad spectrum of thyroid phenotypes as illustrated in Tables 1 and Table 2 [1-5]. Accordingly, careful clinical assessment, particularly in cases of antibody-negative goiter, may suggest DH and prompt further biochemical and molecular evaluation.

**Table 1:** **Genes associated with thyroid dysgenesis [** 1-5 **]**

Gene (OMIM)	Cytogenetic location	Thyroid phenotype	Associated features	Mode of transmission
***TSHR*** **(603372)**	14q31.1	Complete resistance to TSH: severe gland hypoplasia with measurable Tg levels (athyreosis); Partial resistance to TSH: GIS CH with isolated hyperthyrotropinemia	TSH resistant	AD/AR
***NKX2-1*** **(600635)**	14q13.3	Variable	“Brain- Thyroid- lung” syndrome: primary CH, infant respiratory distress syndrome (IRDS), choreoathetosis	AD
***FOXE1*** **(602617)**	9q22.33	Thyroid agenesis or thyroid ectopy	Bamforth-Lazarus syndrome: Cleft palate, choanal atresia, spiky hair, hypoplastic bifid epiglottis	AR
***PAX8*** **(167415)**	2q14.1	Thyroid hypoplasia or thyroid ectopy	Urogenital tract defects (rare)	AD
***NKX2-5*** **(600584)**	5q35.1	GIS, variable hypothyroidism	Congenital heart malformations	Unknown
**GLIS3** **(610192)**	9p24.2	GIS	Permanent neonatal diabetes, renal cystic dysplasia, cholestasis, progressive liver fibrosis, congenital glaucoma; elevated TSH/Tg levels upon L-T4 treatment	AR
***JAG1*** **(601920)**	20p12.2	Variable orthotopic hypoplasia	Alagille syndrome type 1 (ALGS1): liver, heart, skeleton, eye and facial defects	AR (incomplete penetrance)
***NTN1*** **(601614)**	17p13.1	Thyroid ectopy	Congenital ventricular septum defect	AD
***BOREALIN*** ***(609977)***	1p34.3	Thyroid ectopy, hemiagenesis, thyroid asymmetry	ND	AD/AR
***TUBB1*** ***(612901)***	20q13.32	Thyroid ectopy, hypoplasia, hemithyroid or asymmetric thyroid gland	Formation of macroplatelets and platelet hyperaggregation	AD
***TBX1*** ***(602054)***	22q11.21	GIS	DiGeorge syndrome with congenital heart malformations	AD

Abbreviations: AD, autosomal dominant; AR, autosomal recessive; CH, congenital hypothyroidism; GIS, gland in situ; OMIM, Online Mendelian Inheritance in Man; Tg, thyroglobulin; TSH, thyroid-stimulating hormone; ND, data.

**Table 2:** Genes associated with thyroid dyshormonogenesis [1-5]

Gene (OMIM)	Cytogenetic location	Thyroid phenotype	Characteristic features	Mode of transmission
***TPO*** **(606765)**	2p25.3	TIOD, severe hypothyroidism, goiter	High serum Tg	AR
***SLC26A4/PDS*** **(605646)**	7q22.3	Thyroid dyshormonogenesis 2B; PIOD, mild-to-moderate hypothyroidism, goiter	Pendred syndrome: sensorineural deafness, enlarged vestibular aqueduct; High serum Tg	AR
***TG*** ***(188450)***	8q24.22	High iodide uptake, variable hypothyroidism, congenital or rapidly growing goiter	Absent or very low serum Tg level	AR
***SLC5A5/NIS*** **(601843)**	19p13.11	Thyroid dyshormonogenesis 1; Absent or low iodide uptake at scintigraphy, variable hypothyroidism, goiter	ND	AR
***DUOX1 DUOX2*** **(606758/606759)**	15q21.1	PIOD or CIOD, goiter, transient or permanent hypothyroidism of variable severity	High serum Tg	AR/AD
**DUOXA2** **(612772)**	15q21.1	PIOD or CIOD, goiter, transient or permanent hypothyroidism of variable severity	High serum Tg	AR
***IYD/DEHAL1*** ***(612025)***	6q25.1	Thyroid dyshormonogenesis 4; Conserved iodide uptake, negative perchlorate discharge test, goiter, variable hypothyroidism	High serum Tg and MIT/DIT concentrations in serum and urine	AR (incomplete penetrance)
***SLC26A7*** ***(608479)***	8q21.3	Goiter, variable hypothyroidism, conserved iodide uptake, partial defect at perchlorate discharge	High serum Tg	AR

Abbreviations: AD, autosomal dominant; AR, autosomal recessive; CIOD, complete iodide organification defect; DIT, diiodotyrosine; MIT, monoiodotyrosine; OMIM, Online Mendelian Inheritance in Man; PIOD, partial iodide organification defect; Tg, thyroglobulin; TIOD, total iodide organification defect; ND, no data.

Hypothyroidism in older children and adolescents is usually caused by autoimmune thyroid disease (Hashimoto thyroiditis), typically associated with thyroid peroxidase (TPO) antibodies. However, some CH may initially present with normal neonatal thyroid-stimulating hormone (TSH) levels and progress to significant hypothyroidism later in life, as seen in some cases of sodium/iodide symporter *(NIS),* iodotyrosine deiodinase *(IYD),* and dual oxidase 2 (*DUOX2*) mutations [5]. This paper describes the clinical cases of three affected individuals from unrelated families, all of whom were homozygous for a likely pathogenic variant in the *IYD* gene and presented with hypothyroidism during adolescence.

**Figure 1:**
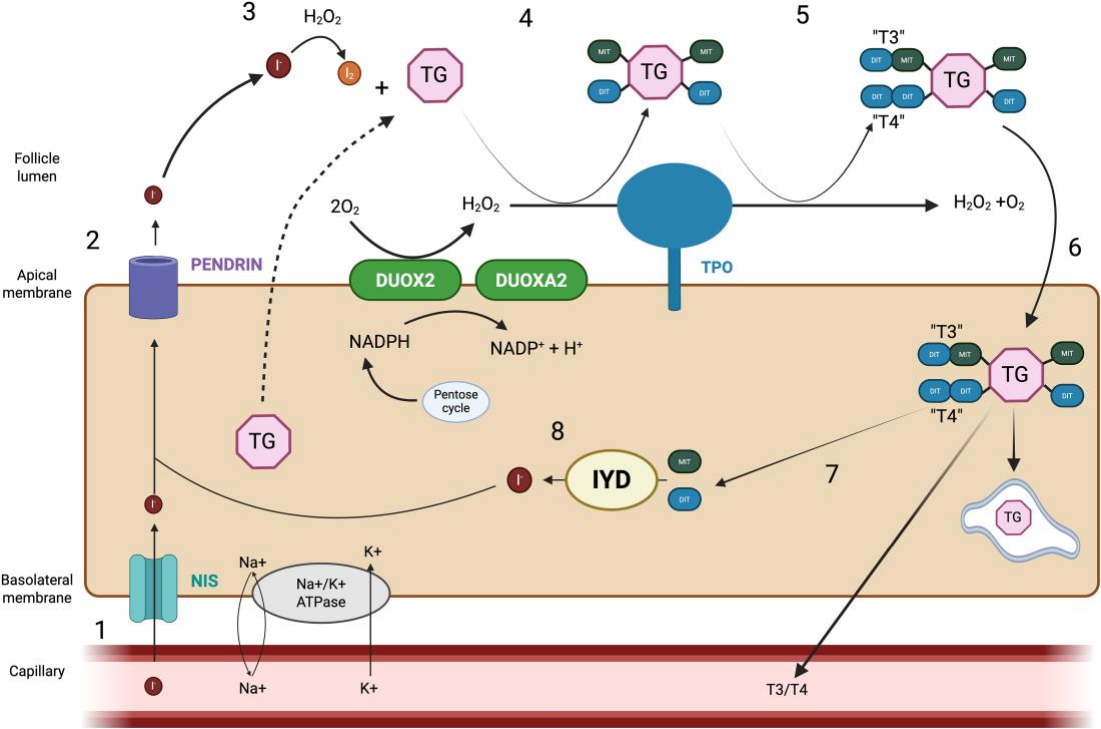
Schematic diagram of a thyrocyte showing the key players involved in thyroid hormonogenesis.^4,6^ Thyroid hormone synthesis is a multistage process which involves the following steps (1) Active transport of iodide from the blood circulation via the NIS which is encoded by *SLC5A5* gene, relying on the electrochemical gradient driven by Na+/K + ATPase pump; (2) The facilitated efflux of iodide into the follicle lumen via an apical anion channel, PENDRIN which is encoded by *SLC26A4* gene; (3) Oxidation of iodine into its active form by the enzyme TPO; (4) Organification of iodine to the tyrosine groups of TG catalyzed by TPO, forming MIT and DIT; (5) Coupling of iodinated tyrosines within TG to iodothyronines (T3 and T4), catalyzed by TPO; (6) Following endocytosis, iodothyronines are cleaved by lysosomal degradation from the TG matrix protein; (7) Iodotyrosines are released and (8) subsequently dehalogenated by IYD allowing “recycling” of iodide for further hormone synthesis. Created in BioRender. Choong, I. (2026) https://BioRender.com/nadz34s. Abbreviations: MIT, monoiodotyrosine; DIT, diiodotyrosine; NIS, sodium/iodide symporter; TG, Thyroglobulin; TPO, Thyroid peroxidase; T4: tetraiodothyronine or thyroxine; T3: triiodothyronine; IYD: iodotyrosine deiodinase. Alt text: Schematic diagram of a thyrocyte illustrating the key transporters, enzymes, and molecular processes involved in thyroid hormone biosynthesis and iodine recycling.

Iodotyrosine deiodinase deficiency (ITDD), a form of DH, is a rare autosomal recessive disorder caused by pathogenic variants in the *IYD* gene. This gene encodes the enzyme IYD, also known as dehalogenase 1 (*DEHAL1*), which is essential for iodine recycling within thyroid cells (thyrocytes). In the absence of functional IYD, monoiodotyrosine (MIT) and diiodotyrosine (DIT) cannot undergo deiodination, resulting in urinary loss of iodotyrosine, as shown in Fig. 1 [4, 6]. Iodine is a key element required for thyroid hormone synthesis, crucial for regulating metabolism and growth [7]. Affected individuals with ITDD typically present with normal thyroid hormone and TSH levels during neonatal screening as the iodine recycling remains sufficient to meet the thyroid hormone synthesis requirement. When demand for thyroxine increases during growth and puberty, the thyroid hormone synthesis becomes inadequate due to the defective recycling of iodine and urinary loss, leading to hypothyroidism and goiter later in childhood or adolescence. Affected individuals are treated with levothyroxine to prevent further complications, which may range from subtle hypothyroid symptoms to severe adverse outcomes. To date, fewer than 20 pathogenic variants in the *IYD* gene have been reported [8], with some developing cognitive defects due to delayed initiation of levothyroxine therapy [1, 7].

## Case presentation

All patients were of South Asian origin and were born to consanguineous parents from unrelated families.

### Case 1

A 13-year-old boy presented with a 6-week history of neck pain and swelling, associated with progressive tiredness and shortness of breath when lying flat. Examination revealed a goiter. He was pubertal (Tanner stage G3). His weight was 83 kg (+2.53 SDS) and height 180.9 cm (+2.08 SDS). His mother has hyperthyroidism, and his father has type 2 diabetes. Parents are first cousins.

### Case 2

A 9-year-old girl presented with a 1-week history of neck swelling and discomfort when swallowing. Over the preceding 4 months, she experienced progressive tiredness, reduced activity levels, worsening mood, hair loss, dry skin, weight gain, and cold intolerance. Pubertal assessment showed Tanner stage 2 breasts and stage 2-3 pubic hair. Her weight was 31.1 kg (−1.08 SDS) and height 135.2 cm (+0.32 SDS). There is no family history of hypothyroidism, but her grandparents have type 2 diabetes. The parents reported possible second-cousin consanguinity. Her younger sister was subsequently diagnosed with hypothyroidism with the same pathogenic variant.

### Case 3

A 15-year-old boy presented with significant weight gain, extreme tiredness, and cold intolerance. He had diffuse goiter and gynecomastia with Tanner stage 3 genitalia and stage 2 pubic hair. His weight was 71.3 kg (+1.60 SDS) and height 169 cm (−0.30 SDS). His maternal uncle had late-onset hypothyroidism. His mother has type 2 diabetes. His parents are distant cousins.

## Diagnostic assessment

All three patients had normal physical examination findings and thyroid function on neonatal screening. However, at the time of clinical presentation, each patient was found to have a diffuse, enlarged goiter on examination. Thyroid function tests (TFTs) revealed elevated TSH and undetectable free T4 levels, consistent with primary hypothyroidism, as summarized in Table 3. Autoimmune screening, including TPO and thyroid-stimulating hormone receptor antibodies (TRAB) were negative in all cases; anti-thyroglobulin antibodies were not routinely assessed in our center.

**Table 3:** Biochemical and ultrasound findings for all patients at initial presentation

Age (years)	Sex	TSH (Normal reference range, 0.35-5.00 µIU/mL; 0.35-5.00 mIU/L)	Free T4 (Normal reference range: 0.70-1.63 ng/dL; 9.00-21.00 pmol/L)	Ultrasound findings of thyroid gland
13.9	Male	**9 µIU/mL (9 mIU/L)**	**<0.39 ng/dL (<5 pmol/L)**	Diffuse enlargement of the thyroid gland without focal nodules or retrosternal extension. The right lobe measured 3.2 × 4.8 × 10.9 cm and the left lobe 3.1 × 5.2 × 10.5 cm, with an isthmus thickness of 1.9 cm.
9.8	Female	**>200 µIU/mL (>200 mIU/L)**	**<0.39 ng/dL (<5 pmol/L)**	Enlarged, hypervascularized thyroid gland with coarse echotexture and no focal abnormalities. The right lobe measured 2.8 × 2.5 × 5.24 cm (volume 18.9 mL) and the left lobe 2.4 × 2.3 × 5.0 cm (volume 14.3 mL). The isthmus thickness was 1.0 cm.
15.5	Male	**>200 µIU/mL (>200 mIU/L)**	**<0.39 ng/dL (<5 pmol/L)**	Enlarged, heterogeneous, hypervascularized thyroid gland without discrete nodules. The right lobe volume was 24 mL and the left lobe volume 18.2 mL.

Abnormal values are shown in bold font. Values in parentheses are International System of Units (SI).

Abbreviations: TSH, thyroid-stimulating hormone; T4, thyroxine; cm, centimeter; mL, milliliter.

Ultrasound examination was also performed to exclude structural abnormalities or malignancy. In our patients, the thyroid gland appeared homogeneously enlarged and hypervascularized, without nodules. No lymphadenopathy was identified. These findings were consistent across our cases (Table 3), demonstrating a morphologically normal but non-functioning gland, a hallmark feature of DH.

Given the absence of thyroid autoantibodies in all three patients, the differential diagnosis included the genetic causes of hypothyroidism or iodine deficiency. However, all patients reported regular consumption of a varied diet including seafood, shellfish, and dairy, making iodine deficiency an unlikely cause. This prompted the need for genetic testing.

Next-generation sequencing (NGS) identified an apparently homozygous variant c.301C >T in the *IYD* gene, which results in an amino acid substitution at the protein level, p.(Arg101Trp). The variant has been previously reported in affected individuals, with in vitro studies demonstrating abolished enzyme activity; hence classified as likely pathogenic under Association for Clinical Genomic Science (ACGS) guidelines and is consistent with DH.

## Treatment

All patients were managed with levothyroxine, a lifelong replacement therapy for permanent primary hypothyroidism. Treatment was initiated at 50 micrograms (mcg) daily and subsequently titrated according to TFTs and clinical response, with maintenance doses ranging from 50-125 mcg/day.

## Outcome and follow-up

All patients were monitored with TFTs every 3-6 months. The goiter subsided with good compliance to levothyroxine therapy. Plasma iodine levels were measured in the first two patients using inductively coupled plasma mass spectrometry (ICP-MS), a highly sensitive method for trace element analysis, as part of their follow-up evaluation.

### Case 1

At 2-month follow-up on levothyroxine 125 mcg/day, the patient remained clinically well and was growing appropriately. Although the goiter was no longer visible on inspection, it remained palpable, likely due to intermittent non-compliance, particularly over weekends. At his most recent follow-up at age 16.3, his TFTs had normalized (TSH 2.37 µIU/mL (2.37 mIU/L) (reference range, 0.35-5.00 µIU/mL; 0.35-5.00 mIU/L); free T4 0.88 ng/dL (11.3 pmol/L) (reference range, 0.70-1.63 ng/dL; 9.00-21.00 pmol/L)).

### Case 2

Between ages 11-13, adherence remained poor with 1-3 missed doses per week, resulting in persistence of goiter and clinical symptoms. Due to irregular TFTs monitoring, dose adjustments were challenging. At follow-up (age 13.5 years) on treatment, the TSH was 1 µIU/mL (1 mIU/L) and free T4 was 1.33 ng/dL (17.20 pmol/L). Despite biochemical normalization at this stage, she exhibited a suboptimal pubertal growth spurt, likely related to previous prolonged periods of poor control. Her final adult height was 150 cm, at the lower end of the parental target height range (father 164 cm; mother 155.4 cm).

### Case 3

At age 15.5, the patient was asymptomatic, reported good compliance, and showed improvement in previously reported hair loss. At age 16.2, in view of low-normal TSH and T4 levels, levothyroxine was stopped for 4 months. Subsequent TFTs confirmed recurrent primary hypothyroidism and reappearance of goiter levothyroxine was reinstated. The patient was discharged to primary care at age 16.8 with well-controlled hypothyroidism (TSH 1.37 µIU/mL (1.37 mIU/L) and free T4 1.08 ng/dL (13.90 pmol/L)).

## Discussion

We report three patients of DH with a pathogenic variant in *IYD*, all of whom presented with clinical features of primary hypothyroidism and goiter during late childhood, around the age of puberty. However, Moreno et al described a younger age of onset [7], which likely reflects differences in residual enzyme activity, genetic heterogeneity, and environmental influences, including iodine intake. Iodine requirements typically increase during late childhood and adolescence, particularly between 9 and 14 years of age [9]. In children with limited dietary iodine intake, pathogenic variants in the *IYD* gene can lower the threshold for the development of hypothyroidism [10]. All our patients had normal thyroid function on neonatal screening, highlighting the delayed presentation that can occur in certain genetic forms of hypothyroidism and the potential diagnostic challenges when early symptoms are subtle.

ITDD was among the earliest recognized forms of DH. The first detailed biochemical characterization of a dehalogenase defect was reported by Stanbury et al in 1955, who demonstrated abnormal release of MIT and DIT into blood and urine using paper chromatography [11]. The missense variant c.301C>T (p.Arg101Trp) identified in our patients was first reported by Moreno et al in 2008 in a consanguineous Turkish family [7], with additional pathogenic variants subsequently described in highly consanguineous families from Scotland and Morocco [12]. Although these historical families are notable, our patients are of South Asian descent and are unrelated to these previously reported families, further supporting the genetic and ethnic heterogeneity of *IYD* deficiency. Consanguinity, observed in all our families, should heighten clinical suspicion for autosomal recessive causes of hypothyroidism, including DH.

From a diagnostic point of view, pathogenic variants in *IYD* should be considered in adolescent patients presenting with a homogeneously enlarged, hypervascularized goiter without micronodularity and negative thyroid autoantibodies. Thyroid ultrasound may serve as a useful adjunct in this context; however, its findings are nonspecific and insufficient to establish a diagnosis of DH in isolation. It may assist in differentiating DH from Hashimoto thyroiditis, which typically demonstrates a heterogeneous and lobulated thyroid gland with micronodules and hypoechogenicity [13]. Emerging research suggests that elevated plasma and urinary levels of MIT and DIT may serve as biochemical markers of *IYD* deficiency, though current clinical assays are limited in sensitivity [7].

Patients with inherited defects in thyroid hormone biosynthesis generally require lifelong levothyroxine therapy. Poor compliance, particularly during adolescence, remains a recognized challenge and was observed in some of our cases, resulting in persistent biochemical abnormalities. Plasma iodine concentrations were obtained after initiation of levothyroxine therapy. In cases of suboptimal thyroxine replacement, plasma iodine levels were low, supporting the role of impaired iodine recycling in disease pathophysiology.

Early recognition and treatment of hypothyroidism are essential to ensure normal growth and neurocognitive development. Given the phenotypic heterogeneity and variable age of onset associated with DH, increased clinical awareness and systematic etiological evaluation using available biochemical and molecular approaches may facilitate the identification of affected individuals. Although thyroid hormone replacement remains the first-line treatment for hypothyroidism, establishing the underlying genetic etiology may provide additional value by informing the recurrence risk and prognosis, enabling targeted family screening and guiding long-term management.

## Learning points

- Genetic causes of hypothyroidism can present later during childhood or adolescence, especially when iodine requirements increase.
- Genetic causes of hypothyroidism should be considered beyond infancy when patients present with antibody-negative hypothyroidism and goiter.
- Consanguinity increases the likelihood of genetic causes of hypothyroidism, particularly DH.
- Ultrasound findings of an enlarged, homogeneous gland without micronodularity can help differentiate adolescent-onset DH from the more common autoimmune hypothyroidism.

## Data Availability

Data sharing is not applicable to this article as no datasets were generated or analyzed during the current study.
